# The Effect of Metformin on Atezolizumab/Bevacizumab Treatment in Patients with Hepatocellular Carcinoma and Diabetes

**DOI:** 10.32604/or.2026.073063

**Published:** 2026-03-23

**Authors:** Andrea Dalbeni, Marco Vicardi, Leonardo A. Natola, Alessandra Auriemma, Bernardo Stefanini, Caterina Vivaldi, Piera Federico, Andrea Polloni, Caterina Soldà, Lorenzo Lani, Ingrid Garajová, Stefano Tamberi, Stefania De Lorenzo, Fabio Piscaglia, Vincenzo Di Maria, Gianluca Masi, Sara Lonardi, Giovanni Brandi, Bruno Daniele, Franco Trevisani, Gianluca Svegliati-Baroni, Laura Schiada, Fabio Marra, Claudia Campani, Ciro Celsa, Giuseppe Cabibbo, Mariangela Bruccoleri, Massimo Iavarone, Leonardo Stella, Francesca R. Ponziani, Tiziana Pressiani, Lorenza Rimassa, Francesco Tovoli, David Sacerdoti

**Affiliations:** 1 Unit of General Medicine C, University of Verona and University and Hospital Trust (AOUI) of Verona, Verona, Italy; 2 Liver Unit, University of Verona and University and Hospital Trust (AOUI) of Verona, Verona, Italy; 3 Section of Innovation Biomedicine-Oncology Area, University of Verona and University and Hospital Trust (AOUI) of Verona, Verona, Italy; 4 Department of Medical and Surgical Sciences, University of Bologna, Bologna, Italy; 5 Unit of Medical Oncology 2, Azienda Ospedaliero-Universitaria Pisana, Pisa, Italy; 6 Medical Oncology Unit, Ospedale del Mare, Napoli, Italy; 7 Oncology Unit, IRCCS Azienda Ospedaliero-Universitaria di Bologna, Bologna, Italy; 8 Oncology 1 Unit, Veneto Institute of Oncology IOV—IRCCS, Padova, Italy; 9 Medical Oncology Unit, University Hospital of Parma, Parma, Italy; 10 Unit of Oncology, Ospedale Santa Maria Delle Croci, Ravenna AUSL Romagna, Ravenna, Italy; 11 Oncology Unit, Azienda USL Bologna, Bologna, Italy; 12 Unit of Internal Medicine, Hepatobiliary and Immunoallergic Diseases, IRCCS Azienda Ospedaliero-Universitaria di Bologna, Bologna, Italy; 13 Unit of Semeiotics, Liver and Alcohol-Related diseases, IRCCS Azienda Ospedaliero-Universitaria di Bologna, Bologna, Italy; 14 Liver Injury and Transplant Unit, Polytechnic University of Marche, Ancona, Italy; 15 Dipartimento di Medicina Sperimentale e Clinica, Università di Firenze, Firenze, Italy; 16 Gastroenterology and Hepatology Unit, Department of Health Promotion, Mother & Child Care, Internal Medicine & Medical Specialties, University of Palermo, Palermo, Italy; 17 Division of Gastroenterology and Hepatology, Foundation IRCCS Cà Granda Ospedale Maggiore Policlinico, Milan, Italy; 18 CRC “A. M. and A. Migliavacca” Center for Liver Disease, Department of Pathophysiology and Transplantation, University of Milan, Milan, Italy; 19 Liver Unit, CEMAD Centro Malattie dell’Apparato Digerente, Medicina Interna e Gastroenterologia, Fondazione Policlinico Universitario Agostino Gemelli IRCCS, Rome, Italy; 20 Humanitas Cancer Center, IRCCS Humanitas Research Hospital, Rozzano, Milan, Italy; 21 Department of Biomedical Sciences, Humanitas University, Pieve Emanuele, Milan, Italy

**Keywords:** Hepatocellular carcinoma, immune checkpoint inhibitors, type 2 diabetes mellitus, metformin, atezolizumab, bevacizumab

## Abstract

**Objectives:**

The combination of atezolizumab plus bevacizumab (A+B) represents one of the standards first-line treatments for unresectable hepatocellular carcinoma (HCC). Metformin has garnered attention for its potential antitumour and immunomodulatory properties beyond glycaemic control. This study aimed to assess metformin’s impact in patients with type 2 diabetes mellitus (T2DM) receiving A+B therapy.

**Methods:**

This retrospective analysis of a prospectively-maintained multicentre database included 523 patients with HCC treated with A+B from the ARTE (Atezolizumab-bevacizumab Real-life Experience for Treatment of Hepatocellular Carcinoma) dataset across 18 Italian centres (May 2020–January 2024). We evaluated objective response rate (ORR), disease control rate (DCR), progression-free survival (PFS), overall survival (OS), and time to progression (TTP) using Cox regression analysis and Inverse Probability of Treatment Weighting (IPTW) to address confounding.

**Results:**

Among 523 patients, 341 (65.2%) did not have diabetes and 182 (34.8%) had T2DM. In the overall population, metformin showed no significant benefit for PFS (HR = 1.15, 95% CI [0.88–1.50], *p* = 0.316) or OS (HR = 1.28, 95% CI [0.94–1.74], *p* = 0.124). In the subgroup with T2DM (N = 180), metformin showed no significant benefit for PFS (HR = 1.41, 95% CI [0.97–2.05], *p* = 0.069), OS (HR = 1.23, 95% CI [0.81–1.86], *p* = 0.333), or TTP (HR = 0.82, 95% CI [0.53–1.26], *p* = 0.363). IPTW analysis confirmed these negative findings.

**Conclusion:**

This study found no evidence of improved outcomes with metformin use in patients with HCC in particular with T2DM receiving A+B therapy. Routine metformin use should not be expected to enhance A+B efficacy based on current evidence.

## 1 Introduction

Hepatocellular carcinoma (HCC) represents the most common primary liver malignancy and remains a major global health challenge [1]. For patients with unresectable disease classified as Barcelona Clinic Liver Cancer (BCLC) stage B or C, systemic therapy with immune checkpoint inhibitors (ICIs) has become the cornerstone of treatment [2]. The combination of atezolizumab, an anti-programmed death-ligand 1 (PD-L1) antibody, plus bevacizumab, a monoclonal antibody targeting vascular endothelial growth factor (VEGF) (A+B), represents one of the current first-line standards of care for unresectable HCC [3]. Other established first-line treatment options include the combination of durvalumab plus tremelimumab (STRIDE regimen) [4–6] and nivolumab plus ipilimumab [7], providing multiple therapeutic approaches for patients with unresectable HCC.

Despite these therapeutic advances, treatment outcomes remain heterogeneous, prompting investigation into factors that may influence immunotherapy efficacy. Patients with type 2 diabetes mellitus (T2DM) represent a significant subpopulation within the broader metabolic liver disease spectrum, yet the specific effects of antidiabetic medications on immunotherapy outcomes remain poorly understood. Metformin, a biguanide antidiabetic agent, has emerged as a compound of particular interest due to its pleiotropic effects extending far beyond glycaemic control [8]. Recent preclinical studies have demonstrated that metformin can directly rescue tumour-infiltrating CD8+ T lymphocytes from hypoxia-induced immunosuppression, thereby enhancing immune checkpoint inhibitor efficacy [9]. This immunomodulatory activity appears independent of metformin’s glucose-lowering effects and involves complex interactions with the tumour microenvironment [10].

Mechanistically, metformin exerts its anticancer effects through multiple pathways. The drug activates AMP-activated protein kinase (AMPK), a central regulator of cellular energy homeostasis, while simultaneously inhibiting the mammalian target of rapamycin (mTOR) signalling pathway, which is frequently dysregulated in HCC [11,12]. This dual action results in reduced cell proliferation, enhanced apoptosis, and altered cellular metabolism. Additionally, metformin downregulates the insulin-like growth factor (IGF) pathway, thereby attenuating mitogenic signals implicated in HCC development and progression [13]. The drug also influences insulin sensitivity and reduces circulating insulin levels, potentially disrupting the pro-tumorigenic metabolic milieu associated with insulin resistance.

Recent systematic reviews and meta-analyses have revealed conflicting evidence regarding metformin’s role in cancer therapy [14,15]. While some studies demonstrate favourable outcomes when metformin is combined with various treatment modalities, others report neutral or even negative effects. This heterogeneity in results has been attributed to differences in patient populations, treatment protocols, study designs, and the specific cancer types investigated. The complexity of these interactions underscores the need for tumour-specific investigations to determine metformin’s therapeutic potential.

Of particular relevance to the current study are metformin’s documented immunomodulatory properties. The drug has been shown to enhance T-cell function, reduce regulatory T-cell populations, and modify the composition of tumour-infiltrating immune cells [16,17]. These effects theoretically position metformin as an ideal adjuvant to immune checkpoint inhibitors, potentially overcoming resistance mechanisms and enhancing therapeutic efficacy. However, the clinical translation of these promising preclinical findings remains incomplete, particularly in the specific context of HCC treatment.

Given this scientific rationale and the clinical importance of optimising outcomes for patients with HCC, particularly those with metabolic comorbidities, we conducted this study using data from the ARTE (Atezolizumab-bevacizumab Real-life Treatment Experience) dataset. Our primary objective was to evaluate the impact of metformin use on treatment outcomes in patients with HCC receiving A+B therapy, with specific focus on the population with T2DM where metformin’s dual metabolic and potential immunomodulatory benefits might be most evident.

## 2 Materials and Methods

### 2.1 Study Design and Population

This retrospective multicentre study analysed prospectively-collected data of patients with unresectable HCC who received A+B across 18 tertiary care centres in Italy between May 2020 and January 2024. Data were prospectively collected and maintained in the ARTE (Atezolizumab-bevacizumab Real-life Experience for Treatment of Hepatocellular Carcinoma) dataset, though the current analysis was conducted retrospectively. Data entries are made biannually using the REDCap platform and are subject to internal consistency checks at the data management center located at IRCCS Azienda Ospedaliero-Universitaria di Bologna. The most recent update to the database was completed in January 2024.

This study protocol was reviewed and approved by the Independent Ethic Committee of the IRCCS Azienda Ospedaliero-Universitaria di Bologna (Protocol Approval No. 811.2022.Os-s.AOUBo), which served as the coordinating center. All other centers received approval from their respective local ethics committees. Written informed consent for treatment and par-ticipation in this study was obtained from all participants. This study adhered to the ethical guidelines of the Declaration of Helsinki.

The inclusion criteria were: (i) radiological and/or histological diagnosis of HCC according to international guidelines [1,18,19]; (ii) unresectable HCC classified as BCLC stage B or C; and (iii) A+B as first-line systemic treatment. Exclusion criteria were age < 18 years and not informed consensus given.

Of 553 patients initially screened, 30 were excluded for unknown diabetes status and 20 for unknown metformin status, yielding 523 patients for the intention-to-treat (ITT) survival analysis. An additional 54 patients were excluded from response analysis due to death or treatment discontinuation before first radiological assessment, resulting in 449 patients for per-protocol response analysis. This exclusion was necessary for response evaluation as radiological assessment is technically impossible in patients who died before imaging; however, all survival analyses included the complete ITT population of 523 patients to maintain analytical integrity.

### 2.2 Data Collection and Definitions

Comprehensive demographic, clinical, and biochemical characteristics were recorded at treatment initiation, including age, sex, Eastern Cooperative Oncology Group Performance Status (ECOG-PS), BCLC stage, antidiabetic treatment regimens, liver disease aetiology, Child-Pugh score, Model for End-Stage Liver Disease (MELD) score, and alpha-fetoprotein (AFP) levels.

MASLD was defined according to recent consensus criteria as hepatic steatosis associated with cardiovascular risk factors (at least 1 out of 5: BMI > 25 kg/m^2^ (BMI > 23 in Asian populations) or waist circumference > 94 cm (men) or 80 cm (women); Fasting serum glucose > 100 mg/dL or HgbA1c > 5.7% or type 2 diabetes or current treatment for type 2 diabetes; Blood Pressure > 130/85 mm/Hg or currently being treated with antihypertensives; Triglycerides > 150 or currently being treated with lipid lowering therapy; HDL cholesterol 40 mg/dL (men) or HDL 50 mg/dL (women) or currently being treated with lipid lowering medication) and alcohol consumption ≤ 2 units daily, while metabolic-alcoholic liver disease (MetALD) was defined as hepatic steatosis with cardiovascular risk factors and 3–4 units daily alcohol intake [20].

T2DM was defined according to international criteria by random blood glucose > 11.1 mmol/L (200 mg/dL) with typical symptoms or glycated haemoglobin (HbA1c) ≥ 48 mmol/mol (≥6.5%) according to international criteria [21].

As part of standard clinical protocols across participating centres, optimal glycaemic control (HbA1c < 75 mmol/mol) was mandatory before ICI initiation following comprehensive endocrinological evaluation to ensure patient safety. This requirement, while potentially limiting generalisability to real-world populations with suboptimal glycaemic control, was implemented as a safety measure given the potential metabolic complications associated with immunotherapy. A+B was administered according to local institutional protocols following multidisciplinary team assessment. Standard dosing comprised atezolizumab 1200 mg intravenously every 3 weeks plus bevacizumab 15 mg/kg every 3 weeks. Treatment continued until disease progression, unacceptable toxicity, or death. Toxicity management, including dose modifications and treatment delays, was conducted according to institutional protocols and product labelling guidelines. Radiological assessments were performed at baseline and every 9–12 weeks using multiphasic contrast-enhanced CT or MRI according to institutional protocols. All assessments were conducted by local radiologists using RECIST criteria version 1.1 [22]. While centralised radiological review was not performed, local radiologists were blinded to clinical data, and any potential bias in progression assessment would have similarly affected all study groups.

Primary outcomes were progression-free survival (PFS), defined as time from first A+B administration to radiological progression or death from any cause, and disease control rate (DCR), defined as the proportion of patients achieving complete response (CR), partial response (PR), or stable disease (SD) as best response.

Secondary outcomes included objective response rate (ORR), defined as the proportion of patients with PR or CR; overall survival (OS), defined as time from treatment initiation to death from any cause; and time to progression (TTP), defined as time from treatment initiation to radiological progression censoring for non-progressive deaths.

### 2.3 Statistical Analysis

Continuous variables were presented as mean ± standard deviation or median with interquartile range according to distribution normality assessed by Shapiro-Wilk test. Categorical variables were expressed as frequencies and percentages. Group comparisons employed Student’s *t*-test or Mann-Whitney U test for continuous variables, and Chi-square or Fisher’s exact test for categorical variables. Survival analyses employed Cox proportional hazards regression with log-rank tests for univariable comparisons. Multivariable Cox regression models assessed independent prognostic factors, with variables showing *p* <0.20 in univariable analysis included in stepwise selection for the final model. Model assumptions were evaluated using Schoenfeld residuals and log-log plots. To address potential confounding by indication, Inverse Probability of Treatment Weighting (IPTW) analysis was performed using propensity scores derived from baseline demographic and clinical covariates. Propensity scores were estimated using logistic regression models, and stabilised weights were applied to balance treatment groups. For multiple comparisons in baseline characteristics between diabetic and non-diabetic groups, Bonferroni correction was applied (α = 0.025). Post-hoc power analysis was conducted to assess the study’s ability to detect clinically meaningful differences in survival outcomes using observed effect sizes, event numbers, and sample sizes. Statistical significance was set at *p* <0.05 for all analyses. All analyses were performed using R software (version 4.2.1)® and SPSS (v.25.0; IBM Corp., Armonk, NY, USA) and STATA® (STATA version 15.0, College Station, TX, USA) software.

## 3 Results

### 3.1 Baseline Characteristic Analysis

A total of 523 patients were included in the final analysis: 341 (65.2%) without diabetes and 182 (34.8%) with T2DM. The majority were male (81.8%) with mean age 68.0 ± 10.5 years. Baseline characteristics are summarised in Table 1.

**Table 1:** Baseline characteristics of the study population (N = 523).

Characteristic	Whole Population (N = 523)	No T2DM (N = 341)	T2DM (N = 182)	*p*-Value
Male sex, n (%)	428 (81.8%)	274 (80.4%)	154 (84.6%)	0.24
Age (years), mean (SD)	68.0 (10.5)	66.5 (11.3)	70.9 (8.3)	<0.001
BMI, mean (SD)	25.8 (4.5)	25.2 (4.2)	27.0 (4.8)	<0.001
HBV, n (%)	97 (18.5%)	72 (21.1%)	25 (13.7%)	0.044
HCV, n (%)	235 (44.9%)	171 (50.1%)	64 (35.2%)	0.001
ALD, n (%)	122 (23.3%)	91 (26.7%)	31 (17.0%)	0.013
MASLD, n (%)	154 (29.4%)	60 (17.6%)	94 (51.6%)	<0.001
MetALD, n (%)	43 (8.2%)	21 (6.2%)	22 (12.1%)	0.029
Hypertension, n (%)	293 (56.1%)	170 (49.9%)	123 (68.0%)	<0.001
Hypercholesterolemia, n (%)	128 (24.7%)	60 (17.7%)	68 (38.0%)	<0.001
Serum creatinine (mg/dL), median (IQR)	0.8 (0.7–1.0)	0.8 (0.7–1.0)	0.9 (0.7–1.1)	0.021
Hb (g/dL), mean (SD)	13.1 (1.8)	13.2 (1.8)	12.9 (1.9)	0.13
ECOG-PS 0, n (%)	361 (69.2%)	243 (71.3%)	118 (65.2%)	0.15
Child-Pugh 5, n (%)	347 (67.9%)	229 (69.4%)	118 (65.2%)	0.40
Number of nodules ≥4, n (%)	274 (52.4%)	178 (52.2%)	96 (52.7%)	0.93
Missing, Number of nodules	37	27	10	N/A
Max diameter (mm), median (IQR)	54.0 (25–100)	60 (27–100)	45 (24.2–80)	0.27
Distribution unilobar, n (%)	184 (37.7%)	118 (37.5%)	66 (38.2%)	N/A
Distribution bilobar, n (%)	253 (51.8%)	163 (51.7%)	90 (52.0%)	N/A
Distribution massive (>50%), n (%)	51 (10.5%)	34 (10.8%)	17 (9.8%)	N/A
Distribution *p*-value	N/A	N/A	N/A	0.94
AFP (ng/mL), median (IQR)	33.9 (5–771.5)	55.5 (5.8–1425.8)	25.4 (4.5–630)	0.054
PVT, n (%)	180 (36.7%)	119 (37.7%)	61 (35.1%)	0.62
Metastasis, n (%)	197 (37.7%)	136 (39.9%)	61 (33.5%)	0.15
Stable disease, n (%)	196 (42.2%)	128 (42.4%)	68 (41.7%)	0.92
Partial response (PR), n (%)	125 (26.9%)	80 (26.5%)	45 (27.6%)	0.83
Complete response (CR), n (%)	29 (6.2%)	17 (5.6%)	12 (7.4%)	0.55
Progressive disease (PD), n (%)	115 (24.7%)	77 (25.5%)	38 (23.3%)	0.65
ORR, n (%)	154 (33.1%)	97 (32.1%)	57 (35.0%)	0.54
DCR, n (%)	350 (75.3%)	225 (74.5%)	125 (76.7%)	0.65
Follow-up (months), median (IQR)	13 (6.1–21.4)	13.4 (6.3–22)	11.9 (5.4–18.8)	0.10
Time to progression (months), median (IQR)	4.9 (2.6–10.9)	4.9 (2.7–10.9)	5.0 (2.2–10.6)	0.73
Death, n (%)	262 (50.1%)	168 (49.3%)	94 (51.6%)	0.65

Note: Detailed table showing demographic, clinical, and tumour characteristics comparing patients without type 2 diabetes mellitus (T2DM) (N = 341) vs. with T2DM (N = 182), including statistical comparisons with Bonferroni-corrected *p*-values where appropriate. Abbreviations: N/A, Not Applicable; BMI, body mass index; HBV, hepatitis B virus; HCV, hepatitis C virus; ALD, alcohol liver disease; MASLD, Metabolic dysfunction–associated steatotic liver disease; MetALD, Metabolic and alcohol liver disease; ECOG-PS, Eastern Cooperative Oncology Group Performance Status; AFP, alfa fetoprotein; PVT, portal vein thrombosis; ORR, Objective response rate; DCR, disease control rate.

Patients with T2DM were significantly older (70.9 ± 8.3 vs. 66.5 ± 11.3 years, *p* < 0.001 after Bonferroni correction) and had higher BMI (27.0 ± 4.8 vs. 25.2 ± 4.2, *p* < 0.001). MASLD was substantially more prevalent in patients with T2DM (51.6% vs. 17.6%, *p* < 0.001), whilst viral hepatitis was more common in patients without T2DM (HCV: 50.1% vs. 35.2%, *p* < 0.001; HBV: 19.1% vs. 9.9%, *p* = 0.044). The adverse events documented during A+B treatments were reported in Supplementary Table S1.

Among 182 patients with T2DM, 181 had known metformin status (Table 2) and were included in subgroup analysis: 93 (51.4%) received metformin monotherapy, 41 (22.7%) insulin-based treatment, and 47 (26.0%) dietary management or other antidiabetic agents.

**Table 2:** Sensitivity analysis of patients, excluding missing values of metformin.

Characteristic	Whole Population (N = 503)	No T2DM (N = 322)	T2DM (N = 181)	*p*-Value
Male sex, n (%)	414 (82.3%)	260 (80.7%)	154 (85.1%)	0.27
Age (years), mean (SD)	68.1 (10.6)	66.6 (11.4)	70.9 (8.3)	<0.001
BMI, mean (SD)	25.8 (4.5)	25.2 (4.2)	27.0 (4.8)	<0.001
HBV, n (%)	93 (18.5%)	69 (21.4%)	24 (13.3%)	0.023
HCV, n (%)	225 (44.7%)	162 (50.3%)	63 (34.8%)	<0.001
ALD, n (%)	116 (23.1%)	85 (26.4%)	31 (17.1%)	0.02
MASLD, n (%)	152 (30.2%)	58 (18.0%)	94 (51.9%)	<0.001
MetALD, n (%)	42 (8.3%)	20 (6.2%)	22 (12.2%)	0.02
Hypertension, n (%)	282 (56.2%)	160 (49.7%)	122 (67.8%)	<0.001
Hypercholesterolemia, n (%)	127 (25.5%)	59 (18.4%)	68 (38.0%)	<0.001
Serum creatinine (mg/dL), median (IQR)	0.8 (0.7–1.0)	0.8 (0.7–1.0)	0.9 (0.7–1.1)	0.036
Hb (g/dL), mean (SD)	13.1 (1.8)	13.2 (1.8)	12.9 (1.9)	0.14
ECOG-PS 0, n (%)	347 (69.1%)	229 (71.1%)	118 (65.6%)	0.17
Child-Pugh 5, n (%)	339 (67.9%)	222 (69.6%)	117 (65.0%)	0.39
Number of nodules ≥4, n (%)	263 (52.3%)	168 (52.2%)	95 (52.5%)	>0.99
Missing, Number of nodules	33	23	10	N/A
Max diameter (mm), median (IQR)	52.0 (25.0–90.0)	57.0 (25.5–100.0)	45.0 (24.0–80.0)	0.55
Distribution unilobar, n (%)	182 (38.6%)	116 (38.7%)	66 (38.4%)	N/A
Distribution bilobar, n (%)	248 (52.5%)	158 (52.7%)	90 (52.3%)	N/A
Distribution massive (>50%), n (%)	42 (8.9%)	26 (8.7%)	16 (9.3%)	N/A
Distribution *p*-value	N/A	N/A	N/A	0.97
AFP (ng/mL), median (IQR)	33.9 (5.0–742.0)	54.0 (5.7–1255.9)	25.2 (4.5–618.1)	0.08
PVT, n (%)	173 (36.5%)	112 (37.2%)	61 (35.3%)	0.69
Metastasis, n (%)	187 (37.2%)	127 (39.4%)	60 (33.1%)	0.18
Stable disease*, n (%)	185 (41.2%)	117 (40.9%)	68 (41.7%)	0.92
Partial response (PR)*, n (%)	123 (27.4%)	78 (27.3%)	45 (27.6%)	>0.99
Complete response (CR)*, n (%)	29 (6.5%)	17 (5.9%)	12 (7.4%)	0.56
Progressive disease (PD)*, n (%)	112 (24.9%)	74 (25.9%)	38 (23.3%)	0.57
ORR*, n (%)	152 (33.9%)	95 (33.2%)	57 (35.0%)	0.76
DCR*, n (%)	337 (75.1%)	212 (74.1%)	125 (76.7%)	0.57
Follow-up (months)*, median (IQR)	13.2 (6.1–21.4)	13.8 (6.2–22.0)	11.9 (5.5–18.9)	0.10
Time to progression (months)*, median (IQR)	4.9 (2.5–10.9)	4.8 (2.6–10.9)	5.0 (2.2–10.6)	0.72
Death*, n (%)	249 (49.5%)	156 (48.4%)	93 (51.4%)	0.58

Note: *excluding patient with missing values of metformin. Abbreviations: BMI, body mass index; HBV, hepatitis B virus; HCV, hepatitis C virus; ALD, alcohol liver disease; MASLD, Metabolic dysfunction–associated steatotic liver disease; MetALD, Metabolic and alcohol liver disease; Hb, Haemoglobin; ECOG-PS, Eastern Cooperative Oncology Group Performance Status; AFP, alfa fetoprotein; PVT, portal vein thrombosis; ORR, Objective response rate; DCR, disease control rate; N/A: Not Applicable.

The general characteristics of the group with diabetes are divided for diabetic treatments are reported in Table 3.

**Table 3:** Baseline characteristics of subgroups with T2DM.

Characteristic	Patients with T2DM (N = 180)	Metformin (N = 93)	Insulin (N = 41)	Dietetic or Others Regiment (N = 46)	*p*-Value
Male sex, n (%)	153 (85.0%)	78 (83.9%)	34 (82.9%)	41 (89.1%)	0.69
Age (years), mean (SD)	70.9 (8.3)	70.7 (7.6)	70.2 (8.4)	71.8 (9.7)	0.63
BMI, mean (SD)	27.0 (4.8)	27.4 (5.0)	26.6 (4.9)	26.5 (4.0)	0.49
HBV, n (%)	24 (13.3%)	7 (7.5%)	5 (12.2%)	12 (26.1%)	0.01
HCV, n (%)	62 (34.4%)	28 (30.1%)	21 (51.2%)	13 (28.3%)	0.04
ALD, n (%)	31 (17.2%)	13 (14.0%)	7 (17.1%)	11 (23.9%)	0.32
MASLD, n (%)	94 (52.2%)	53 (57.0%)	20 (48.8%)	21 (45.7%)	0.41
MetALD, n (%)	22 (12.2%)	14 (15.1%)	4 (9.8%)	4 (8.7%)	0.57
Hypertension, n (%)	122 (68.2%)	69 (75.0%)	26 (63.4%)	27 (58.7%)	0.11
Hypercholesterolemia, n (%)	68 (38.2%)	42 (45.7%)	12 (29.3%)	14 (31.1%)	0.11
Serum creatinine, mean (SD)	1.0 (0.4)	0.9 (0.3)	1.0 (0.5)	1.0 (0.4)	0.15
Hb (g/dL), mean (SD)	13.0 (1.9)	12.8 (1.8)	12.9 (2.0)	13.3 (1.9)	0.35
HbA1C, mean (SD)	49.7 (5.3)	49.1 (5)	49.5 (5.2)	49.4 (5)	0.63
ECOG-PS 0, n (%)	117 (65.4%)	66 (71.0%)	24 (58.5%)	27 (60.0%)	0.37
Child-Pugh 5, n (%)	116 (64.8%)	65 (69.9%)	21 (51.2%)	30 (66.7%)	0.23
Number of nodules ≥ 4 n (%)	94 (52.2%)	50 (53.8%)	21 (51.2%)	23 (50.0%)	0.92
Missing (Number of nodules)	10	6	1	3	N/A
Max diameter (mm), median (IQR)	45.0 (24.0–80.0)	48.5 (22.8–88.5)	44.5 (30.0–74.5)	40.0 (22.0–76.0)	0.93
Distribution unilobar, n (%)	66 (38.6%)	32 (36.4%)	16 (40.0%)	18 (41.9%)	N/A
Distribution bilobar, n (%)	89 (52.0%)	49 (55.7%)	21 (52.5%)	19 (44.2%)	N/A
Distribution massive (>50%), n (%)	16 (9.4%)	7 (8.0%)	3 (7.5%)	6 (14.0%)	N/A
Distribution *p*-value	N/A	N/A	N/A	N/A	0.68
AFP (ng/mL), median (IQR)	25.4 (4.6–623.0)	29.5 (4.8–603.6)	25.0 (5.3–1437.0)	25.4 (3.2–279.6)	0.46
PVT, n (%)	61 (35.5%)	30 (33.7%)	18 (45.0%)	13 (30.2%)	0.34
Metastasis, n (%)	60 (33.3%)	36 (38.7%)	8 (19.5%)	16 (34.8%)	0.08
Stable disease, n (%)	68 (41.7%)	35 (43.2%)	13 (34.2%)	20 (45.5%)	0.54
Partial response (PR), n (%)	45 (27.6%)	19 (23.5%)	13 (34.2%)	13 (29.5%)	0.45
Complete response (CR), n (%)	12 (7.4%)	6 (7.4%)	4 (10.5%)	2 (4.5%)	0.63
Progressive disease (PD), n (%)	38 (23.3%)	21 (25.9%)	8 (21.1%)	9 (20.5%)	0.81
ORR, n (%)	57 (35.0%)	25 (30.9%)	17 (44.7%)	15 (34.1%)	0.34
DCR, n (%)	125 (76.7%)	60 (74.1%)	30 (78.9%)	35 (79.5%)	0.81
Follow-up (months), median (IQR)	11.9 (5.4–18.4)	10.7 (4.8–19.2)	10.3 (6.2–16.3)	15.0 (6.8–21.0)	0.40
Time to progression (months), median (IQR)	5.0 (2.2–10.6)	5.4 (2.3–11.8)	3.1 (2.0–7.2)	6.0 (2.2–10.9)	0.56
Death, n (%)	93 (51.7%)	51 (54.8%)	17 (41.5%)	25 (54.3%)	0.34

Note: This table compares baseline characteristics across metformin, insulin, and dietary or other regimens. Abbreviations: BMI, body mass index; HBV, hepatitis B virus; HCV, hepatitis C virus; ALD, alcohol liver disease; MASLD, Metabolic dysfunction–associated steatotic liver disease; MetALD, Metabolic and alcohol liver disease; Hb, Haemoglobin; ECOG-PS, Eastern Cooperative Oncology Group Performance Status; AFP, alfa fetoprotein; PVT, portal vein thrombosis; ORR, Objective response rate; DCR, disease control rate; N/A: Not Applicable.

**Figure 1:**
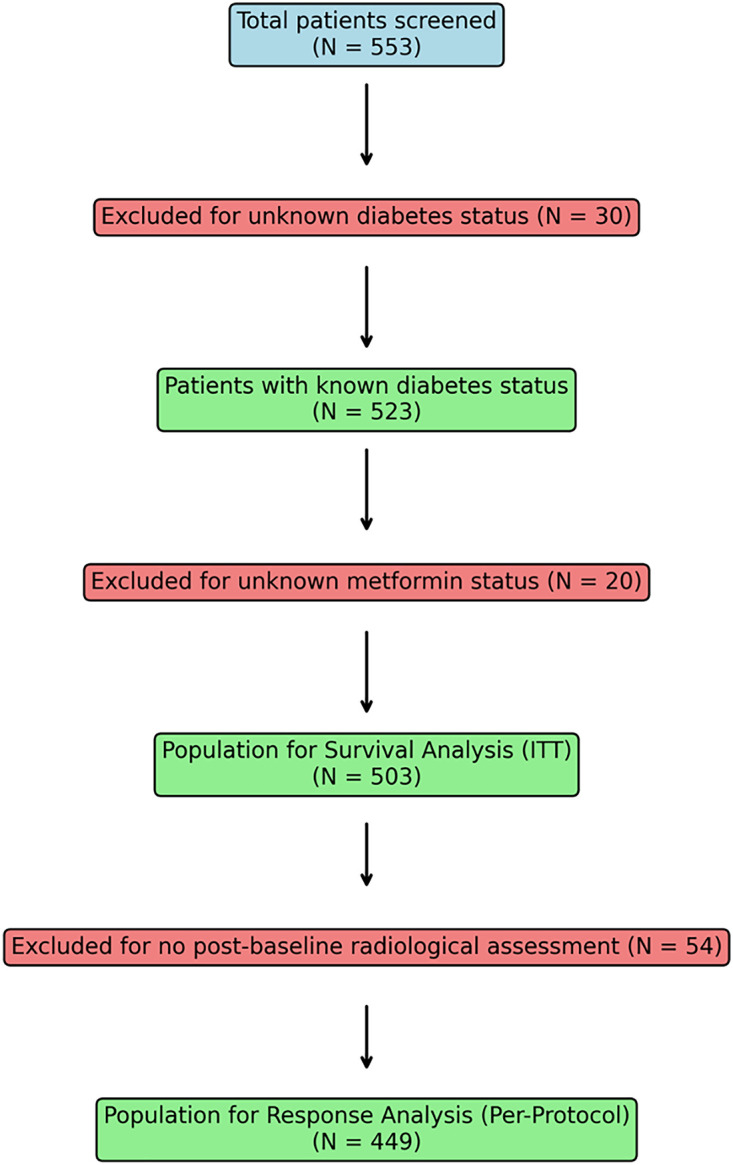
Patient disposition flowchart showing selection process from initial screening (N = 553) to final analysis populations.

In particular, the metformin group showed optimal glycaemic control at baseline similar to other treatment groups (HbA1c: 49.1 ± 5.0 vs. 49.5 ± 5.2 mmol/mol, *p* = 0.63). Fig. 1 presents the comprehensive patient selection flow chart, detailing the progression from initial screening (N = 553) through final analysis populations, clearly showing exclusions and the populations used for different analyses.

**Figure 2:**
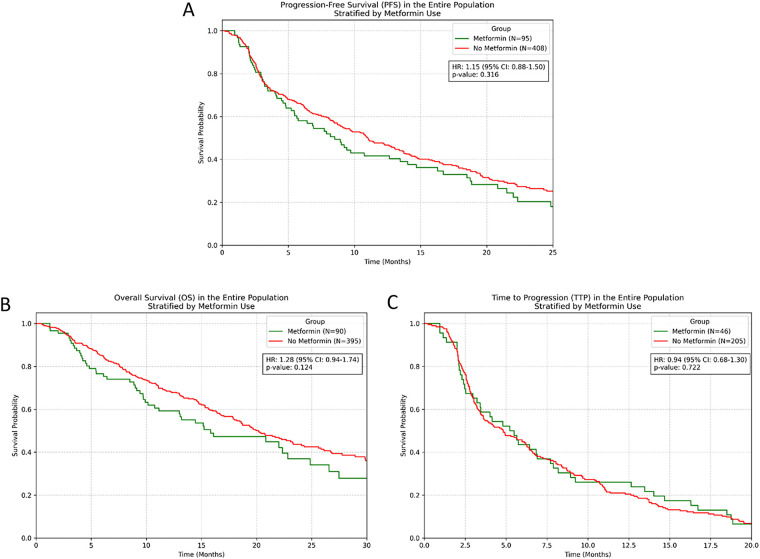
Kaplan-Meier survival curves for entire population stratified by metformin use. (**A**) PFS; (**B**) OS; (**C**) TTP.

In the entire cohort, metformin users demonstrated no significant difference in survival outcomes compared to non-users. PFS analysis revealed HR = 1.15 (95% CI [0.88–1.50], *p* = 0.316), while OS analysis showed HR = 1.28 (95% CI [0.94–1.74], *p* = 0.124). Similarly, TTP analysis demonstrated no significant difference (HR = 0.94, 95% CI [0.68–1.30], *p* = 0.722) (Fig. 2).

**Figure 3:**
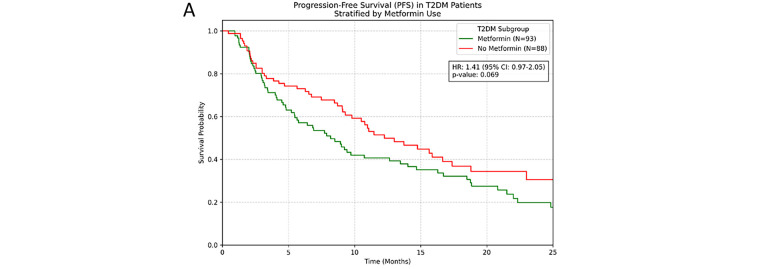

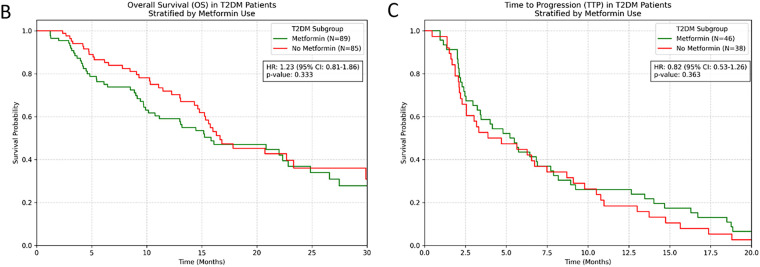
(Kaplan-Meier survival curves for patients with T2DM stratified by metformin use. (**A**) PFS; (**B**) OS; (**C**) TTP).

Treatment response rates were comparable between groups. In the response-evaluable population, ORR was 26.3% in metformin users vs. 23.8% in non-users (*p* = 0.58), whilst DCR was 73.7% vs. 71.2% respectively (*p* = 0.64). Within the population with T2DM (N = 180), contrary to our initial hypothesis, metformin treatment showed a numerical trend towards shorter PFS, though this did not reach statistical significance (HR = 1.41, 95% CI [0.97–2.05], *p* = 0.069). OS analysis revealed no significant difference between metformin and non-metformin groups (HR = 1.23, 95% CI [0.81–1.86], *p* = 0.333). TTP analysis similarly showed no significant benefit with metformin use (HR = 0.82, 95% CI [0.53–1.26], *p* = 0.363) (Fig. 3).

Response rates in the subgroup with T2DM showed no clinically meaningful differences: ORR was 30.9% with metformin vs. 44.7% and 34.1% without metformin (insulin and dietetic or others drugs) (*p* = 0.34), and DCR was 74.1% vs. 78.9% and 79.5%, respectively (*p* = 0.81).

### 3.2 Multivariable and IPTW Analysis

Cox multivariable analysis identified no significant association between metformin use and clinical outcomes in either the overall population or subgroup with T2DM (Tables 4 and 5).

**Table 4:** Hazard ratios (HRs) for progression in the subgroup with T2DM from multivariable Cox regression analysis.

Characteristic	Univariable Analysis			Multivariable Analysis		
	HR	95%CI	*p*-Value	HR	95%CI	*p*-Value
Age	1.02	1.00, 1.05	0.054	N/A	N/A	N/A
Sex (Male)	0.93	0.54, 1.62	0.80	N/A	N/A	N/A
BMI > 25	1.19	0.80, 1.77	0.39	1.15	0.76, 1.74	0.52
HBV	1.50	0.81, 2.75	0.19	N/A	N/A	N/A
HCV	0.66	0.43, 1.02	0.06	0.65	0.41, 1.05	0.07
ALD	0.88	0.52, 1.48	0.63	N/A	N/A	N/A
Serum creatinine	0.91	0.48, 1.74	0.78	N/A	N/A	N/A
Child Pugh ≥ 7	1.27	0.73, 2.19	0.40	1.07	0.60, 1.90	0.83
ECOG-PS ≥ 1	1.26	0.84, 1.88	0.26	N/A	N/A	N/A
Max diameter	1.00	1.00, 1.00	0.27	1.00	1.00, 1.00	0.13
Nodules ≥ 4	0.90	0.61, 1.33	0.59	N/A	N/A	N/A
PVT	1.18	0.79, 1.77	0.40	N/A	N/A	N/A
Metastases	1.33	0.88, 2.02	0.17	N/A	N/A	N/A
Metformin	1.35	0.91, 1.99	0.14	1.29	0.87, 1.93	0.20
AFP	1.00	1.00, 1.00	0.011	N/A	N/A	N/A

Note: This table shows univariable and multivariable HRs with 95% confidence intervals (CIs) for the expanded dataset. Abbreviations: BMI, body mass index; HBV, hepatitis B virus; HCV, hepatitis C virus; ALD, alcohol liver disease; ECOG-PS, Eastern Cooperative Oncology Group Performance Status; PVT, portal vein thrombosis; AFP, alpha-fetoprotein; N/A: Not Applicable.

**Table 5:** Hazard ratios (HRs) for death in the subgroup with T2DM from multivariable Cox regression analysis.

Characteristic	Univariable Analysis			Multivariable Analysis		
	HR	95%CI	*p*-Value	HR	95%CI	*p*-Value
Age	1.01	0.99, 1.04	0.30	N/A	N/A	N/A
Sex (Male)	0.78	0.41, 1.49	0.45	N/A	N/A	N/A
BMI > 25	1.32	0.84, 2.08	0.23	N/A	N/A	N/A
HBV	1.51	0.77, 2.94	0.23	1.71	0.85, 3.45	0.13
HCV	0.65	0.40, 1.06	0.08	N/A	N/A	N/A
ALD	1.04	0.59, 1.83	0.88	N/A	N/A	N/A
Serum creatinine	0.84	0.40, 1.73	0.62	N/A	N/A	N/A
Child Pugh ≥ 7	2.38	1.31, 4.34	0.005	2.40	1.23, 4.69	0.01
ECOG-PS ≥ 1	1.34	0.85, 2.10	0.20	N/A	N/A	N/A
Max diameter	1.00	1.00, 1.00	0.053	1.00	1.00, 1.00	0.62
Nodules ≥ 4	0.86	0.56, 1.34	0.50	N/A	N/A	N/A
PVT	1.49	0.96, 2.33	0.07	N/A	N/A	N/A
Metastases	1.16	0.72, 1.86	0.53	1.22	0.76, 1.97	0.41
Metformin	1.25	0.80, 1.93	0.32	1.29	0.82, 2.05	0.27
AFP	1.00	1.00, 1.00	0.001	1.00	1.00, 1.00	<0.01

Note: This table shows univariable and multivariable HRs with 95% confidence intervals (CIs) for the expanded dataset. Abbreviations: BMI, body mass index; HBV, hepatitis B virus; HCV, hepatitis C virus; ALD, alcohol liver disease; ECOG-PS, Eastern Cooperative Oncology Group Performance Status; PVT, portal vein thrombosis; AFP, alpha-fetoprotein; N/A: Not Applicable.

In the cohort with T2DM, metformin showed no independent prognostic value for progression (HR = 1.29, 95% CI [0.87–1.93], *p* = 0.20) or survival (HR = 1.29, 95% CI [0.82–2.05], *p* = 0.27). IPTW analysis using propensity score weighting confirmed these findings and demonstrated consistency with the multivariable Cox regression results. For the subgroup with T2DM, IPTW-adjusted hazard ratios were: PFS: HR = 1.34 (*p* = 0.14), OS: HR = 1.27 (*p* = 0.28), and TTP: HR = 0.88 (*p* = 0.58). These results demonstrate that the absence of metformin benefit is robust across different analytical approaches. Sensitivity analysis excluding patients with missing follow-up data confirmed the robustness of our findings, with consistent results across all endpoints (Supplementary Table S2).

## 4 Discussion

Our large retrospective multicentre analysis found no evidence supporting improved clinical outcomes with metformin use in patients with HCC receiving A+B therapy, including those with T2DM. These findings contrast with the compelling biological rationale and promising preclinical data suggesting synergistic effects between metformin and immunotherapy. Our results align with the recent findings of Kang et al., who similarly reported no benefit of metformin in a large cohort of patients with HCC receiving various systemic treatments, including A+B, despite the fact that no specific analyses had been conducted on the population with diabetes [23]. This consistency across independent datasets strengthens the evidence against routine metformin use for enhancing immunotherapy efficacy in HCC.

However, our findings differ from studies in other malignancies where metformin has shown promise when combined with ICIs. In non-small-cell lung cancer, Afzal et al. reported improved clinical outcomes in patients receiving concurrent metformin and ICIs [24]. Smith et al. demonstrated enhanced response to ICIs across lung cancer, particularly in obese patients, with metformin combination therapy [25]. Similarly, Afzal et al. showed efficacy benefits in patients with metastatic melanoma treated with metformin plus anti-PD-1/anti-CTLA-4 therapy [26]. However, these studies were limited by small sample sizes, heterogeneous populations, retrospective designs, and a lack of rigorous control for confounding variables. The conflicting evidence in the literature reflects the complexity of metformin’s anticancer mechanisms and their tissue-specific manifestations. Recent systematic reviews have highlighted significant heterogeneity in study designs, patient populations, and outcome measurements across metformin-cancer studies [27,28]. This variability has made definitive conclusions about metformin’s therapeutic utility challenging and underscores the importance of tumour-specific investigations. The absence of clinical benefit in our study, despite preclinical rationale, highlights the complexity of translating laboratory findings to clinical practice.

Metformin’s immunomodulatory effects include enhancement of CD8+ T-cell memory formation, reduction of regulatory T-cell populations, and modulation of the tumour microenvironment [29,30]. Recent studies have demonstrated that metformin can directly counteract hypoxia-induced immunosuppression in tumour-infiltrating lymphocytes, potentially enhancing ICI efficacy [31]. However, these effects may be dose-dependent, timing-dependent, or require specific tumour microenvironmental conditions to manifest clinically. In HCC, where liver dysfunction and altered hepatic metabolism are common, the expected benefits of metabolic modulation through metformin may be attenuated or require different therapeutic approaches than those used for diabetes management. Additionally, the mandatory requirement for optimal glycaemic control in our study may have minimised metabolic differences between treatment groups, potentially reducing the differential effects of metformin. Moreover, missing data on important variables including diabetes duration, severity of complications, previous glycemic control history, exact medication dosing regimens, and duration of metformin therapy may have influenced results and represents an inherent limitation of retrospective analyses. Given the established evidence that immunotherapy efficacy is consistent across different HCC aetiologies, including metabolic-associated liver disease, our findings suggest that metformin’s potential benefits may not be sufficient to overcome the inherent challenges of treating HCC with current immunotherapy approaches. The liver’s unique immunological environment, particularly in the setting of chronic liver disease and metabolic dysfunction, creates distinct challenges for immunotherapy [32]. The complex metabolic milieu of the liver may influence metformin’s bioavailability and therapeutic effects in ways that differ from other cancer types, potentially explaining the variable responses observed clinically [33].

Our study has several important limitations that must be acknowledged. The retrospective design inherently limits causal inference and introduces potential unmeasured confounding despite IPTW adjustment. The exclusion of patients without follow-up imaging for response analysis and patients with unknow metformin status administration may have introduced selection bias towards patients with better performance status and treatment tolerance. Missing data on important variables including diabetes duration, severity of complications, previous glycaemic control history, exact medication dosing regimens, and duration of metformin therapy may have influenced results. The single-country design conducted across Italian tertiary care centres limits international generalisability to other healthcare systems with different patient populations, treatment protocols, and supportive care measures. The lack of centralised radiological review may have introduced assessment variability, though this limitation likely affected all treatment groups equally. Additionally, the requirement for optimal glycaemic control before treatment initiation, while clinically appropriate, may have reduced the potential to observe differential effects between metformin and non-metformin groups. This protocol requirement could limit the applicability of our findings to real-world populations with suboptimal diabetes control.

Despite these limitations, our study provides valuable real-world evidence in a substantial patient population treated at multiple specialised centres. Strengths include the large sample size, multicentre design, comprehensive data collection from a prospectively maintained database, implementation of rigorous statistical methods including IPTW analysis, and transparent reporting of negative results. The inclusion of detailed power analysis provides important context for interpreting our findings and planning future studies.

The negative findings of our study have important implications for clinical practice and future research. Currently, there is insufficient evidence to support routine metformin use for enhancing immunotherapy efficacy in patients with HCC, even in those with T2DM where the drug is already indicated for diabetes management. Treatment decisions regarding metformin should continue to be based primarily on standard diabetic care indications rather than anticipated oncological benefits. However, the biological rationale for metformin-ICI synergy remains compelling, and our findings should not be interpreted as definitive evidence against this therapeutic strategy. The significant limitations in statistical power mean that modest but clinically relevant benefits cannot be excluded, but neither confirmed. Additionally, the possibility that specific patient subsets, optimal dosing strategies, or different timing of metformin initiation might reveal benefits requires further investigation.

Future research should prioritise adequately powered prospective randomised controlled trials specifically designed to evaluate metformin’s role in combination with ICIs in HCC, although it is believed that other antidiabetic regimens such as the combination of metformin with GLP-1 may be of greater interest today.

## 5 Conclusions

It was found that no evidence of improved clinical outcomes with metformin use in patients with unresectable HCC receiving A+B therapy, including in the subpopulation with T2DM. These findings suggest that routine metformin use should not be expected to enhance A+B efficacy based on current evidence. However, the compelling biological rationale for metformin-ICI synergy, combined with the limitations of our study design, warrant further investigations in larger and more diverse cohorts.

## Supplementary Materials



## Data Availability

The data that support the findings of this study are not publicly available due to privacy reasons but are available from the corresponding author upon reasonable request.

## Supplementary Materials

The supplementary material is available online at https://www.techscience.com/doi/10.32604/or.2026.073063/s1.
