# Supplementary material for: The Effect of Metformin on Atezolizumab/Bevacizumab Treatment in Patients with Hepatocellular Carcinoma and Diabetes
Source: Oncol Res. 2026 Mar 23;34(4):16. doi: 10.32604/or.2026.073063 (PMC13040334; doi:10.32604/or.2026.073063)
Supplement: Supplementary file 1 [file OncolRes-34-73063-s001.docx]

**Supplementary Table S1:** Adverse Events (AEs) in the whole population and in the subgroup.

| **Characteristic** | **Whole Population (N = 523)** | **No T2DM (N = 341)** | **T2DM (N = 182)** |
| --- | --- | --- | --- |
| Discontinuation due to AEs, n (%) | 50 (9.0%) | 33 (9.7%) | 13 (7.1%) |
| Death due to Toxicity, n (%) | 7 (1.3%) | 3 (0.9%) | 4 (2.2%) |
| Severe AEs (Grade ≥ 3), n (%) | 63 (11.4%) | 42 (12.3%) | 20 (11.0%) |
| Any other AE reported, n (%) | 255 (46.1%) | 155 (45.5%) | 85 (46.7%) |
| Decompensated Cirrhosis (Ascites), n (%) | 67 (12.1%) | 41 (12%) | 21 (11.5%) |
| Hepatic Encephalopathy, n (%) | 5 (0.9%) | 2 (0.6%) | 3 (1.6%) |
| New onset Hypertension, n (%) | 139 (25.1%) | 82 (24.0%) | 47 (25.8%) |
| Heart Failure, n (%) | 3 (0.5%) | 2 (0.6%) | 1 (0.5%) |
| Arterial Thromboembolism, n (%) | 13 (2.4%) | 9 (2.6%) | 4 (2.2%) |
| Venous Thromboembolism, n (%) | 19 (3.4%) | 13 (3.8%) | 5 (2.7%) |

**Supplementary Table S2.** IPTW (Inverse Probability of Treatment Weighting) analysis results showing propensity score-weighted analyses for all endpoints.

| **Analysis** | **Comparison** | **Endpoint** | **HR** | **95% CI** | ***p*-value** |
| --- | --- | --- | --- | --- | --- |
| Whole population | Metformin *vs.* No Metformin | PFS | 1.12 | 0.82, 1.52 | 0.47 |
|  | Metformin *vs.* No Metformin | OS | 1.24 | 0.87, 1.77 | 0.23 |
|  | Metformin *vs.* No Metformin | TTP | 0.88 | 0.60, 1.29 | 0.49 |
| T2DM Subgroup | Metformin *vs.* No Metformin | PFS | 1.34 | 0.90, 2.00 | 0.14 |
|  | Metformin *vs.* No Metformin | OS | 1.27 | 0.82, 1.97 | 0.28 |
|  | Metformin *vs.* No Metformin | TTP | 0.88 | 0.55, 1.40 | 0.58 |
|  | Insulin Only *vs.* Diet Only | PFS | 1.56 | 0.87, 2.79 | 0.14 |
|  | Metformin *vs.* Diet Only | PFS | 1.48 | 0.94, 2.35 | 0.09 |
|  | Insulin Only *vs.* Diet Only | OS | 1.22 | 0.63, 2.35 | 0.56 |
|  | Metformin *vs.* Diet Only | OS | 1.37 | 0.80, 2.33 | 0.25 |
|  | Insulin Only *vs.* Diet Only | TTP | 1.77 | 0.78, 4.01 | 0.17 |
|  | Metformin *vs.* Diet Only | TTP | 1.26 | 0.77, 2.06 | 0.35 |

Abbreviations: ORR, objective response rate; DCR, disease control rate; PFS, progression-free survival; OS, overall survival; TTP, time to progression; HR, hazard ratio; CI, confidence interval.
